# Compassionate Care with Autonomous AI Humanoid Robots in Future Healthcare Delivery: A Multisensory Simulation of Next-Generation Models

**DOI:** 10.3390/biomimetics9110687

**Published:** 2024-11-11

**Authors:** Joannes Paulus Tolentino Hernandez

**Affiliations:** 1 Nursing Faculty, Generic Bachelor of Science (GBS) Degree Program, Helene Fuld College of Nursing, New York, NY 10035, USA; joannes.tolentinohernandez@helenefuld.edu or jphernandezrn@gmail.com; 2 Advanced SpaceLife Research Institute (ASRI), Cape Canaveral, FL 32920, USA; 3 Aerospace Medical Association (AsMA), Alexandria, VA 22314, USA; 4 Sigma Theta Tau International Honor Society of Nursing—Alpha Zeta Chapter, Indianapolis, IN 46202, USA; 5 International Association for Human Caring, Westwood, MA 02090, USA; 6 American Nurses Association, Silver Spring, MD 20910, USA; 7 Global Society for Philippine Nurse Researchers, Inc. (GSPNRI), Malate, Metro Manila 1004, Philippines

**Keywords:** AI, robotics, HRI, posthumanism, nursing, compassionate care, quantum computing, neuromorphic computing, agent-based modeling, multisensory simulation, data sonification, TRETON, Martha Rogers, Jean Watson, TCCN, nurse robots, space robots, robot proxies, aerospace nursing

## Abstract

The integration of AI and robotics in healthcare raises concerns, and additional issues regarding autonomous systems are anticipated. Effective communication is crucial for robots to be seen as “caring”, necessitating advanced mechatronic design and natural language processing (NLP). This paper examines the potential of humanoid robots to autonomously replicate compassionate care. The study employs computational simulations using mathematical and agent-based modeling to analyze human–robot interactions (HRIs) surpassing Tetsuya Tanioka’s TRETON. It incorporates stochastic elements (through neuromorphic computing) and quantum-inspired concepts (through the lens of Martha Rogers’ theory), running simulations over 100 iterations to analyze complex behaviors. Multisensory simulations (visual and audio) demonstrate the significance of “dynamic communication”, (relational) “entanglement”, and (healthcare system and robot’s function) “superpositioning” in HRIs. Quantum and neuromorphic computing may enable humanoid robots to empathetically respond to human emotions, based on Jean Watson’s ten caritas processes for creating transpersonal states. Autonomous AI humanoid robots will redefine the norms of “caring”. Establishing “pluralistic agreements” through open discussions among stakeholders worldwide is necessary to align innovations with the values of compassionate care within a “posthumanist” framework, where the compassionate care provided by Level 4 robots meets human expectations. Achieving compassionate care with autonomous AI humanoid robots involves translating nursing, communication, computer science, and engineering concepts into robotic care representations while considering ethical discourses through collaborative efforts. Nurses should lead the design and implementation of AI and robots guided by “technological knowing” in Rozzano Locsin’s TCCN theory.

## 1. Introduction

The integration of artificial intelligence (AI) and robotics in healthcare raises ethical and safety concerns regarding reliability, quality, and empathy [1]. Effective communication is crucial, as demonstrated by the Pepper robot, which utilizes advanced signal processing for speech and emotional recognition [2]. In Japan, nurses act as intermediaries between humanoid robots and patients instead of robots becoming proxies for caregiving. This conservative approach may hinder the acceptance of direct robotic nursing care [3]. Traditional nursing philosophies often exclude robotics, challenging existing care paradigms, as “caring” involves complex cognitive functions and emotional validation [4]. For humanoid robots to be perceived as caring, they must incorporate advanced mechatronic design and natural language processing (also known as NLP) [5].

As robots evolve within society, they will be integral to human experiences [6], with future AI capable of organizing information to acquire knowledge [7,8]. This identifies the need for intuitive models of robots in nursing practices and a clear definition of “humanness” in robots to promote user acceptance. This is supported by theories such as Technological Competency as Caring in Nursing (TCCN) by Locsin [9], which posits that “caring” for individuals is achieved through “technological knowing” to utilize advanced technologies (e.g., robots), and the Transactive Relationship Theory of Nursing (TRETON) by Tanioka [10], which opens the use of humanoid robots in nursing care.

Despite skepticism about robots in caregiving, the collaboration between a nurse and a robot presents a chance to reframe nursing within a “posthumanist” context that embraces nonhuman caring agencies [11] as an extension of “Nursing”. However, with the pursuit and installation of autonomous AI, humanoid robots will gain greater traction and become rivals to human competencies.

The forthcoming humanoid robots will anticipate needs, recognize emotions, and respond empathetically, exceeding the capabilities of current TRETON-based models (e.g., the Pepper robot). However, these future technological marvels will not be limited to the field of nursing but are expected to be transdisciplinary, including medicine, computer science, robotics, psychology, and other sciences, and the arts.

We may also foresee the “Next-Generation Models” venturing into outer space, surpassing earlier models in capability and functionality. Fong and Nourbakhsh [12] stated that NASA (the National Aeronautics and Space Administration) had one-way communication that limited effectiveness. Additionally, Hambuchen, Marquez, and Fong [13] identified challenges such as communication latencies, limited bandwidth, and an insufficient technical analysis of robotic systems. Despite these challenges, AI in optimizing human–robot interaction (HRI) remains largely untapped. Robotic systems are invaluable for remote interaction and resource processing. There is an urgency for effective HRI in healthcare delivery to astronauts. Autonomous AI humanoid robots are ideal for long-distance space missions, like Mars, where Earth-based mission control shifts to mission support. Furthermore, nurse-designed robots could pave the way for their inclusion in the space industry and the establishment of aerospace nursing education, research, practice, and leadership.

Use of computer simulations can expand our predictive abilities beyond the constraints of human imagination, especially in complex scenarios [14]. While our innate ability to foresee outcomes is valuable, computer-based tools can better simulate intricate interactions, such as those between humans and robots in caregiving contexts. Computer simulations, produced as multisensory experiences, are exhibited in plots with data sonification. The latter gives agent-based modeling its auditory dimension to complement the representation of the nonlinear and unpredictable dynamics of agent interactions during their “entanglement”, where information exchange occurs. The humanoid robot’s capacity to “know” what to sense and learn emerges when it engages in “superpositioning” with the healthcare system as a care provider. Operating at an autonomous AI level grants it comparable autonomy—more importantly, the ability to make clinical judgments—and situational awareness through perceiving its environment and interacting with other agents, both human and nonhuman.

The data sonification component of the simulation provides objective methods (i.e., mapping data parameters) and intuitive methods (i.e., interpreting how and why it sounds as it does) for inquiring about HRIs. The auditory equivalent of each visualization simplifies the identification of patterns, trends, and anomalies. It supports a meaningful exploration of complex datasets and understanding of abstract concepts like quantum mechanics in robot behavior. This is particularly valuable for evaluating robot system designs and their integration into human-centric environments by audibly representing communication patterns and system dynamics, and an opportunity for “fine-tuning” the model for reliable, reproducible, and valid simulations when they become out of sync.

### Aims

This paper discusses the application of humanoid robot technology in healthcare by identifying key concerns from the literature, including the technological advancements of robotics through AI, the impact of robotics on human caring and nursing philosophy, and the potential outcomes of autonomous AI humanoid robots in nursing as a progression in contemporary practice using multisensory simulation models.

## 2. Salient Discussions in Humanoid Robotics

The literature collectively enumerates the potential benefits, challenges, and ethical considerations of deploying humanoid robots in care settings. While the benefits are promising—such as enhanced patient engagement, support for healthcare workers, and (re)solutions to workforce shortages—the challenges and ethical concerns are substantial. These include user acceptance, technological limitations, and the fundamental question of whether humanoid robots can deliver genuine care. The future of social robots in healthcare hinges on addressing these challenges through rigorous research, ethical scrutiny, and policy development to ensure their effective and responsible integration into care settings.

### 2.1. Replicating Compassionate Care and Ethical and Policy Considerations

“Compassion” is defined as a “warm-hearted wish to see others free from suffering”, emphasizing its benevolent nature and focus on alleviating suffering. It is a multidimensional construct with affective components, involving feelings of warmth towards others, and cognitive components, encompassing awareness of their difficulties. This perspective is supported by research in affective neuroscience (Ashar et al., 2016; Dahl et al., 2016) and Tibetan Buddhist teachings (Dalai Lama, 2005), as noted by Ash et al. [15]. Autonomous AI humanoid robots should replicate “compassionate care” by combining (a) heartfelt emotional connection with the person being cared for, (b) cognitive awareness of that person’s suffering, and (c) a genuine desire to alleviate that suffering. These aspirational features in robotics are groundbreaking, but the pursuit of achieving symmetry with human intelligence (from an analogue to a homologue) and affective ability through communication could represent a significant leap in design. 

Lee et al. [16] examined the role of care robots in Korean nursing services, identifying key tasks such as “measuring/monitoring” and “mobility/activity” where robots could reduce workload, while also raising concerns about malfunctions and reduced human interaction. Diño et al. [17] reviewed healthcare robotics for older adults, discussing the need for user-centered design and technological competency among nurses, and mentioning that current systems are less anthropomorphic with limited emotional capabilities. Nieto Agraz et al. [18] classified 133 nursing care robotic projects, identifying “use cases” (user–system interactions for problem solving) like logistics and patient autonomy, and gaps in practical deployment and HRI.

Broadbent [19], Johanson, Ahn, and Broadbent [20], and van Kemenade, Konijn, and Hoorn [21] stressed the importance of designing robotic systems that can communicate empathy and understanding. The ethical implications of these technologies are outlined by van Wynsberghe [22,23,24] and Coghlan [25]. They advocated for robust frameworks for development and deployment. Applications of robotics in elderly care have been examined by Mordoch et al. [26], Turja et al. [27], and Getson and Nejat [28], concerning social companionship and assistance with daily living activities. The impact of robotics on the healthcare workforce, particularly how it may alter occupational roles and required skills, was discussed by Papadopoulos et al. [29] and Strudwick, Wiljer, and Inglis [30]. Additionally, researchers like Lekova et al. [31] and Zaier [32] have investigated robotics in rehabilitation settings.

Recent research conducted by El-Gazar et al. [33] and Soljacic et al. [34] articulated progress in the use of AI for clinical decision making, which contributes to both diagnostic precision and personalized treatment strategies. Despite these advancements, significant issues still persist. Privacy continues to be a major issue, alongside biases inherent in algorithms. Additionally, there is a pressing need for updated legal and regulatory frameworks to respond to these evolving circumstances, according to Bertolini and Arian [35] and Terry [36].

The social and cultural implications of these technologies have also been examined by Lewandowska-Tomaszczyk and Wilson [37] and Čaić et al. [38], with the necessity of considering diverse cultural contexts in their adoption. Future research, as indicated by Axelsson, Spitale, and Gunes [39] and Lancaster [40], must tackle technological limitations, improve patient acceptance, and ensure the ethical and sustainable use of AI and robotics in healthcare settings.

Yuan et al. [41] conveyed the importance of embedding care ethics into robot design to meet the needs of both care recipients and caregivers, as well as address the limitations of current robot technology in achieving human-like attentiveness and emotional responsiveness. Barcaro, Mazzoleni, and Virgili [42] focused on the moral dimensions of robotic caregiving, detailing the ethical implications of AI applications in healthcare and the need for a “care-centered value sensitive design” (CCVSD) approach. Mijares and Chan [43] analyzed the growing role of robotics in healthcare, raising concerns about the impact on jobs and the patient–caregiver relationship, while presenting methods to program ethical behavior into robots. Vallverdú and Casacuberta [44] first argued for “technological competency”, and Locsin [9] later deemed it essential to care, asserting that quality healthcare depends on expert skills and the need for new technologies to enhance, not diminish, care quality. The long-term effects of robot caregivers on human caregiving skills and care quality need further study.

Sætra [45] proposed policy frameworks to evaluate the use of social robots, focusing on the structure, process, and outcome of care. Locsin and Ito [46] and Locsin et al. [47] delved into the philosophical and ethical implications of using robots in nursing care, questioning whether robots can truly provide “caring” in the human sense. Kipnis et al. [48] and Persson et al. [49] discussed the ethical challenges of HRI in healthcare settings, with the need to respect the autonomy and dignity of care recipients and ensure that robots do not replace indispensable human contact and relationships.

Therefore, the humanistic or anthropomorphic expression of compassion must be a pillar in the development of robotics and AI, while ethical considerations and policy frameworks should govern their implementation and regulation, particularly in the management and security of patients’ and clients’ data and information, and in the training of technology operators, largely by nurses. HRI, as a phenomenon, is not a decoupled reality in light of the “caring field”.

### 2.2. Potential Benefits and Applications

Tanioka T. et al. and Tanioka R. et al. [2,50,51,52,53] and Hernandez [54] conceptualized the roles of humanoid robots in the healthcare industry. The former conducted an extensive inquiry into the capabilities of these automatons to support nursing care, particularly in the context of an aging population in Japan, nursing shortages, and designing robots that can express empathy and engage in meaningful interactions with patients. The latter proposed that conversational robots, in the form of nurse chatbots, can be deployed to meet the telehealth demand for chronic disease self-management support. Conversely, Cano et al. [55] and Trainum et al. [56] focused on the design and implementation of social robots for children with autism spectrum disorder to promote communication and engagement.

The studies by Osaka et al. [57] and Miyagawa et al. [58] assessed the use of robots in dementia care, demonstrating their ability to provide cognitive stimulation and emotional support to patients. Hung et al. [59,60] confer about social robots as companions and motivators for children undergoing medical treatments. Abdi et al. [61] and Guemghar et al. [62] studied the use of socially assistive robots for older adults that could contribute to promoting independence and reducing feelings of loneliness.

### 2.3. User Acceptance and Interaction

User acceptance is a critical factor in the successful implementation of social robots. David et al. [63] found that the acceptability of social robots is influenced by factors such as the intended use, degree of interaction, and user characteristics. Their review indicates a generally positive attitude towards social robots, although ambivalence and resistance are also observed. Betriana et al. [64,65] identified generational differences in the appreciation and utilization of healthcare robots, with “Generation Z” (born between 1997 and 2012) showing more engagement compared to “Baby Boomers” (born between 1946 and 1964). Hurst et al. [66] and Triantafyllidis et al. [67] studied the acceptance of social robots in various healthcare settings, including hospitals and home care, and recommended tailored approaches to promote acceptance across diverse user groups.

### 2.4. Implementation Discourses and Limitations

Despite the potential benefits, the deployment of social robots faces several challenges. González-González et al. [68] pointed out not only technological limitations but also user acceptance issues, integration challenges with existing healthcare workflows, and scalability concerns. Furthermore, they noted the lack of standardized terminology and a consolidated research community around social robots in healthcare, suggesting a need for more extensive collaboration and consensus building. Many studies, including those by Sætra [45] and Locsin and Ito [46], are theoretical and lack empirical data, limiting their applicability in real-world settings.

### 2.5. Impact on Healthcare Professionals

The impact of social robots on healthcare professionals is another important theme found in the literature. Dawe et al. [69] and Hernandez [54] discussed how humanoid robots can impact nurses and healthcare providers by balancing supply and demand, with both support and job displacement risks, in line with Mijares and Chan [43] and Vallverdú and Casacuberta [44]. Morgan et al. [70] and Soriano et al. [71] believe that robots can support healthcare workers and improve patient care, but this should be achieved through careful integration to augment the work rather than replace human labor.

### 2.6. Future Directions

Looking to the future, Ohneberg et al. [72] conveyed the significance of multi-disciplinary collaborations at the intersections of AI, human–computer interaction, and healthcare in robot development. Trainum et al. [56] placed great value on the user-centered design of robots for older adults. Kyrarini et al. [73] and Kitt et al. [74] analyzed the technical limitations of developing these robots. Furthermore, there is a great opportunity to optimize AI for HRI and robot designs in the healthcare delivery for astronauts (and space travelers), from Fong and Nourbakhsh [12] and Hambuchen, Marquez, and Fong [13]. However, there is a lack of studies proposing a Level 4 HRI model and the “techno-ethical” disruptions (in terms of the design, use, and distribution of technology) that “autonomous AI humanoid robots” (which operate independently as caregivers) could introduce to healthcare and society, either as a solution or as a source of uncertainty that may lead to new norms in clinical practice.

## 3. Methods

The research design employs computational simulations using mathematical and agent-based modeling (ABM) to analyze patient–robot–healthcare interactions. This approach incorporates stochastic elements and quantum-inspired concepts, running simulations over 100 iterations to examine complex behaviors without requiring extensive real-world data. ABM simulates complex adaptive systems through interacting autonomous agents, allowing macroscopic behaviors to emerge from microscopic interactions. It effectively models heterogeneous agents and adaptive behaviors by integrating parameterized behavioral rules and decision-making heuristics. Monte Carlo methods, typically involving 100 or more iterations, address stochasticity and explore potential outcomes. This methodology, as demonstrated by Wu et al. [75] and Van Voorn et al. [76], helps achieve quasi-steady states, establish comparative baselines, and mitigate stochastic fluctuations in outputs. The selection of 100 iterations strikes a balance between computational feasibility and statistical robustness, which improves the validity and generalizability of ABM results while providing insights into emergent phenomena and complex interdependencies that analytical methods may overlook.

### 3.1. Conceptualizations of Humanoid Robots and Healthcare Systems

The adaptive capabilities of robots, including emotion-based and memory-based adaptations, are essential for effective human-robot interaction (HRI) [77]. The level of fidelity in terms of emotion generation in humanoid robots is openly debatable. Whether the perceived realism of artificial emotions, such as the emulation of empathy, is genuine or not falls within a “posthumanistic” dimension and requires the acceptance of a standard qualification. Therefore, emotions should be quantized when processing them as information. Emotional communication, in which the experiences between entities or agents can persist even at a distance, can be framed as “spooky action at a distance” or “quantum entanglement” in the context of Albert Einstein’s quantum theory [78]. The development of “pseudo-empathic” humanoid robots will create a deep sense of connection between robots and the humans they care for, likely being perceived by humans as “compassionate care”, regardless of space (onsite or virtual) and time (during and after the care). The perspective aligns with nurse theorist Martha Rogers’ view on the interconnectedness of individuals and their environments [79].

**Figure 1:**
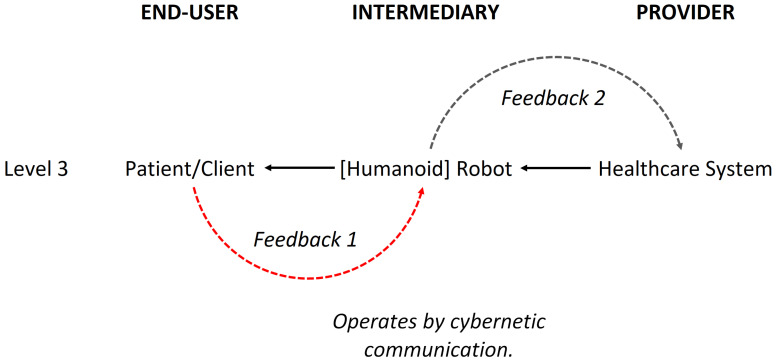
Interpretation of Tanioka’s [10] model according to cybernetic HRI communication [92].

**Figure 2:**
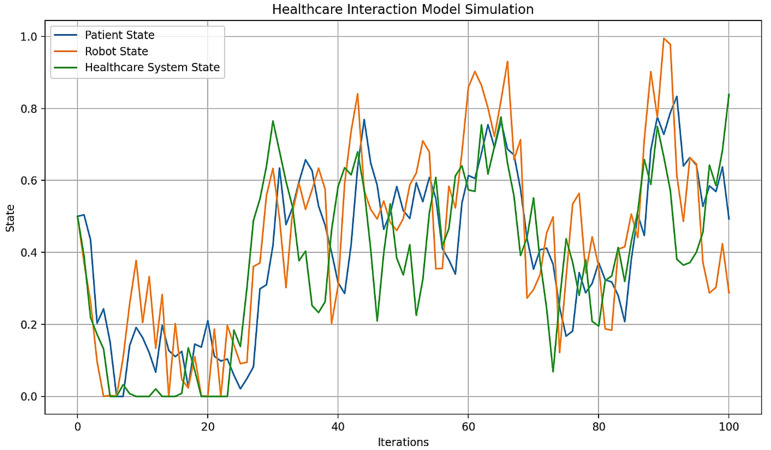
Communication in “Level 3” HRI [92].

**Figure 3:**
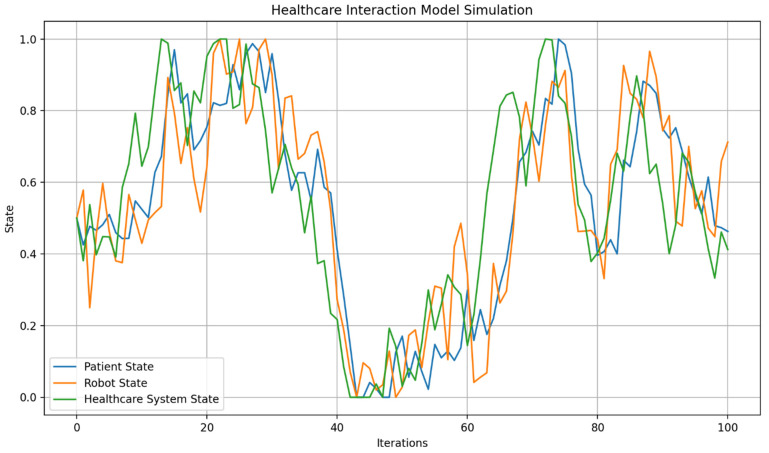
Model validation for “Level 3” HRI [92].

**Figure 4:**
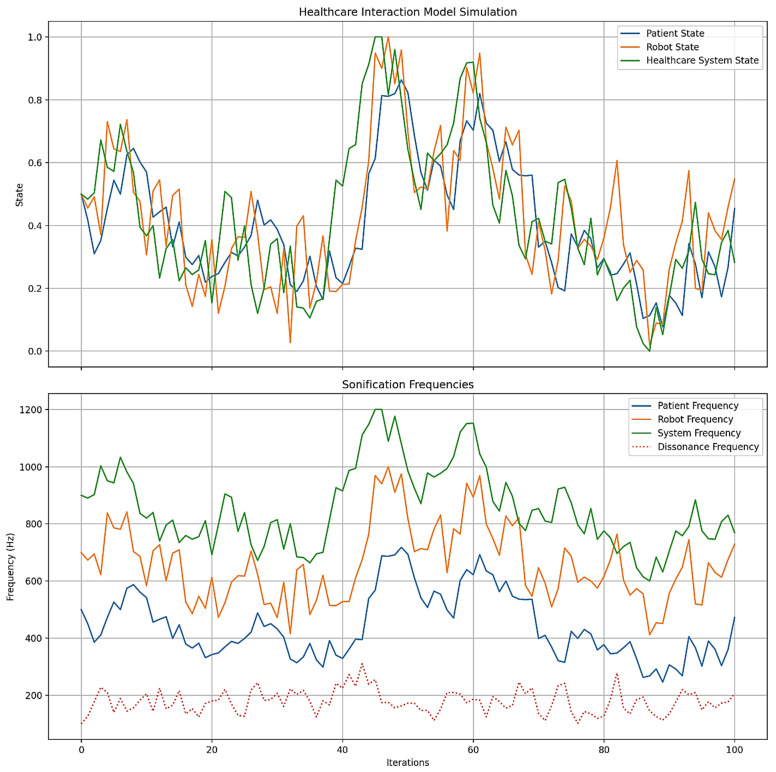
The representation of dissonance with “Level 3” HRI [92]. (Download the file at https://github.com/jphernandezrn/Data-Sonification-Human-Robot-Interaction (accessed on 25 August 2024).

**Figure 5:**
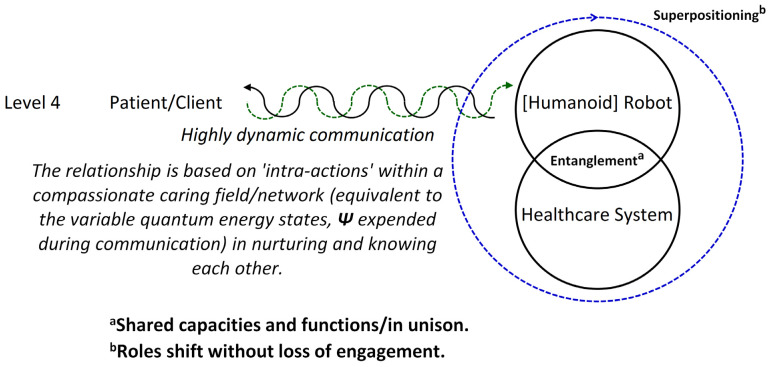
The representation of Level 4 HRI. (Note: The mathematics in quantum communication is referenced from Yuan and Cheng [94], when discussing fidelity).

Humanoid robots can improve their caregiving and social capabilities through adaptability and anthropomorphism, recognizing human-like qualities in emotions and interactions with others [80,81]. Translating the expression of human compassion (both verbal and nonverbal) into AI and healthcare robots algorithmically is essential for their communication and understanding of the symbolic language of “caring”, thus making it humanizing. Attempts to humanize robotic interactions in caregiving contexts are evident in the transition from Figures 1–5.

Through quantum mechanics, represented by the wave function (Ψ) (Figure 5), autonomous AI humanoid robots can embody complex states of interaction and communication with patients and healthcare networks. The principles of “entanglement” and “superposition” permit these robots to achieve multimodal communication simultaneously and exhibit “posthumanistic intelligence”—a form of intelligence that transcends human limitations and is based on collaboration between humans and AI. This capability contributes to their recognition and response to human emotions with high sensitivity and precision, theoretically resulting in compassionate behaviors.

**Figure 9:**
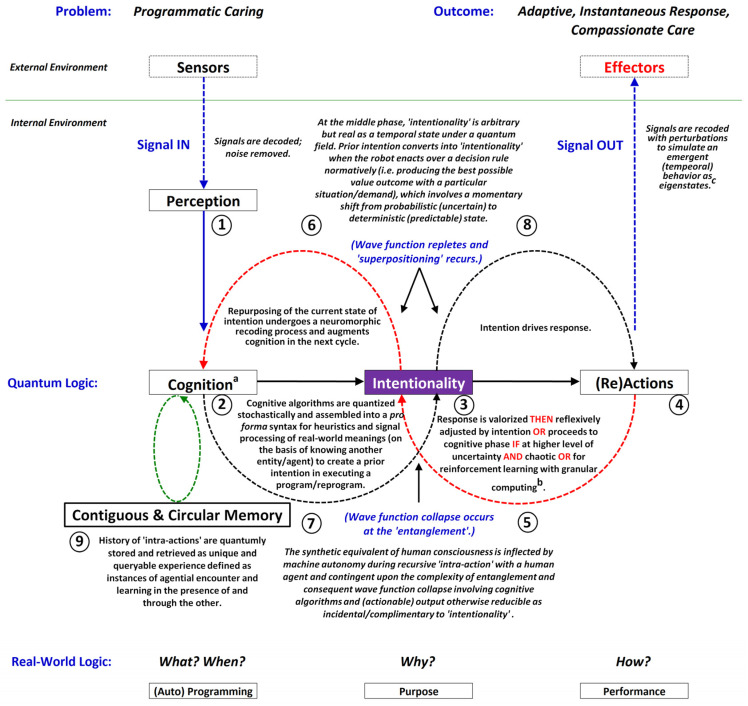
An intuitive, self-regulating, and agile robot system architecture through steps 1–9. Note: ^a^ Information processing must be dynamic, symbolically instantiated (unsupervised), and evolving (unbounded materially) through ^c^ “state transition” (the humanoid robot’s conditions based on actions or events). Unbounded transitions refer to a system’s capacity for an unlimited number of transitions between states, often occurring when the conditions for transitions are not strictly defined or when the system can respond to a wide variety of inputs. In the real world, second-order cybernetics [99] should allow the operation of artificial cognition that is fluid and capable of co-creating knowledge within the healthcare network. ^b^ Alternatively, it can involve the construction and decomposition of “information granules” (the chunks of information) [95], applicable to both algorithmic (deductive) and non-algorithmic (inductive and abductive) computing using quantum logic. This process evolves through machine learning with quantum logic.

To be socially relevant, robot designs must consider cultural variations among end-users to align with diverse values and expectations. For instance, healthcare workers from “collectivist cultures” [82] prioritize group needs and may focus on safety and malfunction protocols. Leveraging quantum algorithms can analyze complex safety scenarios and predict malfunctions by evaluating multiple variables simultaneously (as insights generated from Figures 5 and 9), allowing autonomous AI humanoid robots to anticipate and mitigate risks. A quantum-enhanced humanoid robot can optimize behavior across cultural frameworks by maintaining cultural response patterns in “superposition”, enabling instant adaptation of safety protocols, communication styles, and risk mitigation strategies based on the user’s cultural context.

Figures 1, 5, and 9 present the scientific progression in the complexity and sophistication of human–robot interaction (HRI) within healthcare systems. Figure 1 depicts Tanioka’s model [10], which is grounded in cybernetic communication theory and establishes fundamental feedback loops between the patient, humanoid robot, and healthcare system, featuring a bidirectional flow of information. Figure 5 advances this framework by introducing the Level 4 HRI model, which includes “dynamic communication”, the ability to adjust in real time to a changing environment, and “entanglement”, the joint attunement in sensing, knowing, and feeling the presence of others, along with metacognitive reflection on one’s thought processes. The model also includes “superpositioning”, which refers to the tendency to exhibit agency in thinking and acting. These concepts suggest a more integrated and flexible interaction paradigm, drawing on quantum mechanics to describe relational dynamics and indicating a shift towards more adaptive and responsive robot systems.

Gielis, Shankar, and Prorok [83] noted the lack of co-design approaches that integrate robotic and communication capabilities, advocating for a “meta-system” to overcome these limitations. They discussed data-driven optimization using machine learning and reinforcement learning for robotic perception—extracting knowledge from sensor data, action, and communication. However, issues such as message dropouts and asynchronous reception are common. New communication paradigms are required for wireless data transmission (to and from humanoid robots) as we transition from 5G to 6G technologies. Current “communication-aware algorithms” also fail to adequately model “communication dependencies” or situations where one communication process depends on another.

Figure 9 shows the robot architecture, including internal processes like signal processing, cognition, and intentionality, supported by quantum logic and stochastic algorithms. This model involves adaptive learning and memory systems for delivering instantaneous and compassionate care. It reflects the evolution from basic feedback mechanisms to cognitive and relational processes, indicating advancements in intelligent communication and multimodal sensing. This progression points to significant theoretical advancements in healthcare robotics, aiming to advance patient care in the future.

The studies by Ehrlich et al. [84], Ezra Tsur and Elkana [85], and Hajizada et al. [86] elucidate applications of neuromorphic computing, which mimics human brain function through spiking neural networks and stochastic information processing. These are applied in assistive technologies, pediatric neurorehabilitation, and object recognition, leading to adaptive AI systems that respond effectively to dynamic real-world conditions. Ehrlich et al. [84] developed a neuromorphic adaptive control system for robotic arms, which has real-time adaptability and will be energy-efficient. Ezra Tsur and Elkana [85] reviewed AI-driven robotics in pediatric neurorehabilitation, concentrating on adaptive behavior and explainable AI. Hajizada et al. [86] introduced a spiking neural network architecture for continual object learning, addressing the issue of “catastrophic forgetting (i.e., previous task knowledge is lost when new task information is incorporated into artificial neural networks). Common themes across these studies include adaptive and continual learning, important for effective AI-driven robotics in nursing care, and the energy efficiency of neuromorphic approaches. Real-world testing, scalability, and trust in autonomous systems require further investigation.

Figure 9 aligns with the themes of neuromorphic computing, adaptive learning, and HRI from Ehrlich et al. [84], Ezra Tsur and Elkana [85], and Hajizada et al. [86]. It includes sensors and perception modules for real-time adaptability, corresponding to Ehrlich et al.’s work on robotic arm control. The cognitive modules using stochastic algorithms connect with the adaptive learning in all three studies, particularly Hajizada et al.’s continual object learning. The circular memory component supports adaptive behavior and learning from interactions, according to Ezra Tsur and Elkana. However, the neuromorphic recoding process introduces challenges related to scalability and complexity. Figure 9 displays real-time adaptability and HRI in the proposed adaptive responses.

Healthcare humanoid robots can embody “humanness” in caring by moving beyond mechanistic responses to provide explicit, anticipatory care through anthropomorphic form, logical thinking, and nonlinear learning (Figure 9). This “humanness” quality involves “intentionality”—interpreting the environment to formulate desired actions [87] and using consciousness to influence wellbeing [88]. Authentic “caring” in autonomous AI humanoid robots involves a dynamic interplay between cognition and action, leading to consciousness through information entropy integration [89]. To emulate “humanness”, these robots must be self-determining, with intent and conscious decision making, respecting human dignity and displaying compassionate care [90,91]. Autonomous AI humanoid robots should help patients feel comforted, similar to human nurses’ healing energy presence.

Out of Figure 9, the term “digital plasticity” is proposed as a key mechanism for “dynamic adaptation and learning” (the ability to adjust behavior in real-time based on environmental changes or user interactions) in autonomous AI humanoid robots during HRIs. Automatons modify their behaviors, decision-making, and learning algorithms based on real-time feedback and user interactions. This is especially important in healthcare, where robots must exhibit “emergent learning” by adapting care strategies and communication to individual patient needs (Figures 14 and 15). By integrating advanced computational methods like neuromorphic computing and quantum-inspired algorithms, robots can simulate human-like cognitive flexibility and parallel processing. This makes it possible for them to refine their “caring” behaviors over time, leading to personalized and empathetic interactions. It also ensures that robots adapt their responses to cultural variations, individual preferences, and specific care needs, which are requisites for effective human-robot interactions in healthcare. This capability supports emotional and memory-based adaptations for effective healthcare delivery, accounting for cultural variations in user interactions.

Tying quantum mechanics to Martha Rogers’ “Science of Unitary Human Beings” (SUHB) offers a profound perspective on AI and robotics in healthcare through the interconnectedness of humans, their environment, and robotic caregivers. The phrase “quantum entanglement” [78] connotes this fundamental connection, aligning with Rogers’ view of individuals as “open energy fields” in constant interaction with their surroundings [7,78]. This perspective views new HRIs as an “entanglement of energy fields” arising from the processing of information between interfacing realities—human and humanoid robot—that transcends physical interactions, similar to those found in virtual care. The essence of “caring” resides in the recollected experience, which includes a longer memory, even in humanoid robots.

The non-local nature of quantum mechanics asserts Rogers’ principle of “pandimensionality”, which views reality as nonlinear and free from spatial or temporal constraints [7,79]. This perspective encourages the development of autonomous systems that operate beyond linear time and space, so algorithms can anticipate patient needs through holistic patterns. The principle of “resonancy” denotes the interconnectedness of systems, while “helicy” pertains to the continuous evolution of HRIs. Together, these principles contribute to the adaptability of autonomous AI humanoid robots. The “wave function collapses” in Level 4 HRI model simulations (Figures 9, 10, and 12) represent sudden shifts in the robot’s decision-making and critical thinking, shaped by intents.

Additionally, nursing care plans that apply Martha Rogers’ model, which includes “pattern appraisal”, “mutual patterning”, and “evaluation”, could be performed systematically and more quickly through quantum computing [79]. AI and robotic caregivers can be designed to perceive and respond to the entirety of a patient’s being, including emotional and spiritual dimensions that are interconnected with their surroundings, in alignment with Rogers’ SUHB [7,79].

### 3.2. Simulations

The computational process was executed using Python 3.11.3 in Microsoft Visual Studio Code 1.91.0. Key libraries included Random for stochastic processes, Numpy for numerical operations, Matplotlib.pyplot for visualization, and Pandas for data manipulation. Pydub handled audio manipulation, while Sklearn supported machine learning with metrics, model selection, and ensemble modules, including the random forest regressor. Scipy.stats was used for a statistical analysis, and Collections.deque for circular memory structures. Functions like “random.uniform”, “np.clip”, “stats.pearsonr”, “mean squared error”, and “cross val score” facilitated simulation, data processing, and model evaluation.

**Figure 6:**
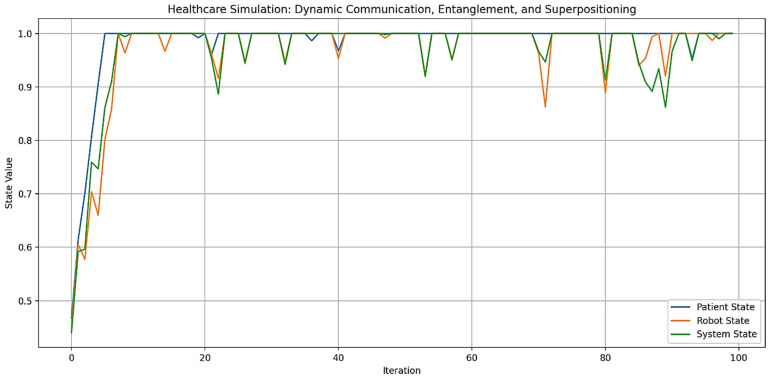
The communication, entanglement, and superpositioning of the three states.

**Figure 7:**
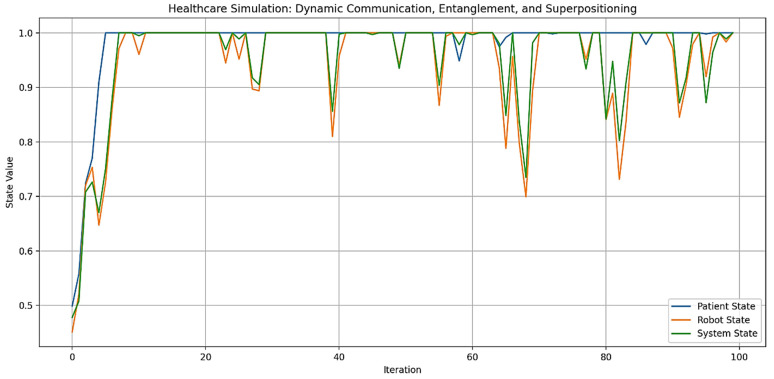
Model validation involving overlapping states.

**Figure 8:**
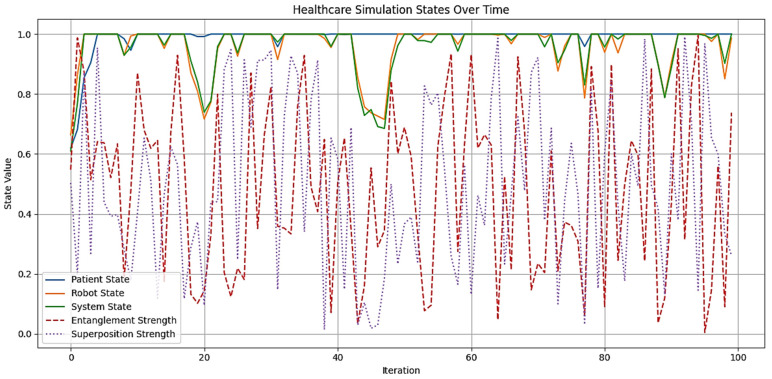
The sonification of frequencies between states exhibiting quantum relationships. (Download the file at https://github.com/jphernandezrn/Data-Sonification-Human-Robot-Interaction).

**Figure 10:**
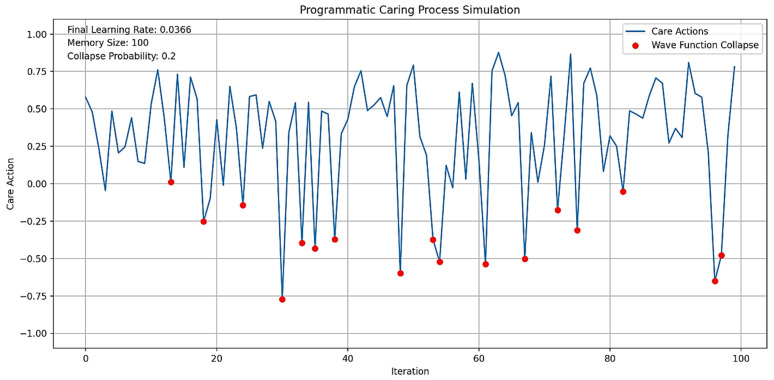
Care actions and intentionality construed from wave function collapse.

**Figure 11:**
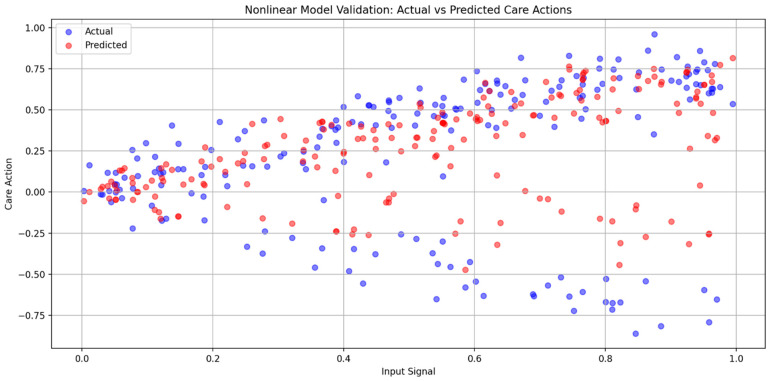
Model validation using machine learning.

**Figure 12:**
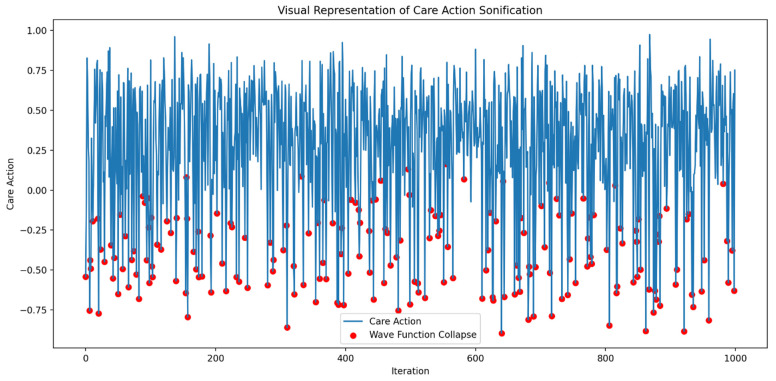
The data sonification of simulated care actions. Download the file at https://github.com/jphernandezrn/Data-Sonification-Human-Robot-Interaction (accessed on 25 August 2024).

Figures 2–4 simulate the information flow among the healthcare system, robot, and patient, utilizing Matplotlib for visualization. The “validate model” function ensured simulation integrity by checking state ranges, correlations, trends, and variability, as shown in Figure 3, while sonification was used to map state values to audio frequencies in Figure 4. Figures 6–8 and 10 introduce quantum concepts such as “entanglement” and “superposition” to simulate the caring process, covering sensor input, robotic perception, cognition, “intentionality” (the intents), action, and learning, with features such as “wave function collapse”. Figure 11 undergoes machine learning validation by training a random forest regressor on simulation data and then validating it using the mean squared error (MSE), *R*^2^, and cross-validation, along with a feature importance analysis. Figure 12 shows a sonification of the data points.

**Figure 13:**
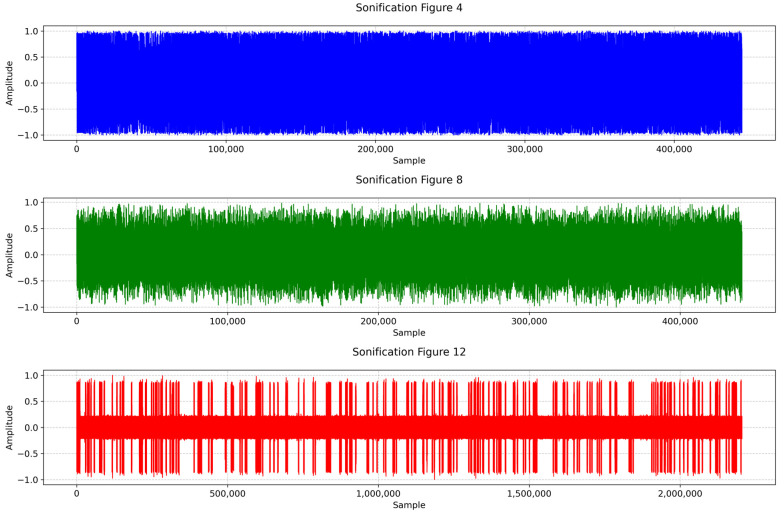
The spectrogram comparison of the three audio files.

Figure 13 uses Pydub, Numpy, and Matplotlib.pyplot to analyze sonification audio files. Audio files are loaded with “AudioSegment.from mp3” and converted to Numpy arrays using “get array of samples”, and then normalized. Waveforms are visualized with “plt.plot” in separate subplots for comparison. The Numpy functions “np.mean” and “np.std” calculate the mean and standard deviation, quantifying central tendency and variability. This combines visual and statistical analyses to understand trends and differences in the sonification data.

The multisensory simulations employed Librosa, Numpy, Matplotlib, and Moviepy. The process begins by loading the audio file from the previous data sonification using “librosa.load”, followed by computing the short-time Fourier transform and converting the spectrogram to decibels. Images (Figures 4, 8, 12, and 19) are read using “plt.imread”. A figure with two subplots displays the image and spectrogram. The animation was created with “FuncAnimation” at 30 frames per second, and the video was saved using the “libx264” codec. The final output combines the animation with the original audio, providing a synchronized audio–visual representation of the simulation data.

The computer circuit simulations (Figures 18 and 19) utilized Numpy, Matplotlib.pyplot, and Random libraries, involving two classes: “NeuromorphicCircuit” and “QuantumEnhancedNeuromorphicCircuit”. These classes process stochasticity values (0 to 1) representing caregiving scenarios. The circuits are initialized with coefficients a, b, and c to compute intentionality using the quadratic function (ax^2^ + bx + c). “NeuromorphicCircuit” handles basic decision making, while “QuantumEnhancedNeuromorphicCircuit” adds quantum-inspired components, including a quantum analog-to-digital converter. Both circuits adaptively learn by adjusting coefficients based on stored input–output pairs. The simulation generates 100–1000 data points to evaluate and compare the output intentionality of classical and quantum-enhanced circuits against input stochasticity.

## 4. Results

Figures 2, 6, and 10 initialize the states of the patient, robot, and healthcare system at 0.5 and run for 100 iterations to create dynamic interactions. Figure 2 simulates information flow and feedback exchanges between the healthcare system, robot, and patient, with random variations of −0.1 to 0.1 for information flow and −0.2 to 0.2 for feedback, constrained within 0 to 1. Interactions influence evolving states over time, causing unpredictability or nonlinearity. Figure 6 introduces “dynamic communication”, “entanglement”, and “superpositioning”, each influencing interaction intensity and state values in healthcare interactions. The states are also clipped to remain within [0, 1] for validity. Figure 10 includes a circular memory that stores 100 experiences, with a learning rate of 1.0 and an uncertainty threshold of 0.5, allowing the system to adapt its care actions, intentions, and outcomes based on past experiences while incorporating randomness to account for real-world dynamics. The choice of 100 iterations balances meaningful trends with computational efficiency, allowing for a robust analysis of agent interactions among patients, robots, and the healthcare system.

Figure 1 is a three-level healthcare interaction framework that utilizes “cybernetic communication (i.e., the Shannon–Weaver model of communication in a circular, self-regulating process) to improve healthcare delivery. It involves three agents: the END-USER (“Patient/Client”), the INTERMEDIARY (“Robot”), and the PROVIDER (“Healthcare System”). Communication flows are depicted with arrows, indicating the direction of information exchange. A solid black arrow represents the flow from the “Healthcare System” to the “[Humanoid] Robot” and then to the “Patient/Client”. Feedback loops are shown by a red dashed arrow (“Feedback 1”) from the “Patient/Client” to the “[Humanoid] Robot” and a black dashed arrow (“Feedback 2”) from the “[Humanoid] Robot” to the “Healthcare System”. Figure 1 positions the robot as an intermediary that facilitates two-way feedback and communication between patients and their healthcare providers within a network system, delivering personalized and accessible healthcare services.

Figure 1 can be represented by the equation $X_{t+1}=\mathrm{clip}\left(\frac{X_{t}+Y_{t}}{2}+\epsilon \right),\;\epsilon ∼U\left(-\alpha ,\alpha \right)$. Here, X and Y are the states of interacting agencies (patient, robot, and healthcare system), *t* is the time step, and ϵ is a random variable. The parameter α is 0.1 for state updates and 0.2 for feedback. The clip function, $clip\left(x\right)=\max\left(0,\min\left(1,x\right)\right)$, keeps state values within [0, 1]. This equation models state updates and feedback, combining deterministic interactions with stochastic variability.

Figure 2 is based on a three-level cybernetic communication system illustrated in Figure 1. This model simulates the information flow from the healthcare system to the robot, communication from the robot to the patient, and feedback loops between the patient and the robot, as well as from the robot back to the healthcare system. After 100 iterations, the final states were “Patient” at 0.4929, “[Humanoid] Robot” at 0.2875, and “Healthcare System” at 0.8390. These results indicate an improvement in the healthcare system’s state, likely due to effective feedback, while the robot’s state decreased, suggesting potential stress or demand on the intermediary. The patient’s state remained relatively stable; however, changes occurred due to the complex interactions within the system. Figure 2 reveals the behavior of agents and the system in a “Level 3” HRI [93], alluding to the quality of care.

Repeated simulations reveal that the robot’s performance, indicated by its logical “state” interval of 0.2875 to 0.8543, exhibits significant fluctuations. This variability indicates the need for improvements in response accuracy, interaction, and overall functionality to ensure a more reliable and efficient experience for patients and clients. Additionally, the patient engagement level, starting at 0.4929, presents an opportunity to increase engagement through personalized verbal and nonverbal interactions. While the healthcare system state can range from 0.5970 to 1.0000, there are still opportunities to streamline processes, improve communication, and strengthen technology integration to better support patient care. Finally, actively incorporating user feedback to refine the robot’s knowledge base and functionality can effectively resolve specific concerns and increase patient or client satisfaction.

Figure 3 is based on the interactions between a patient, a humanoid robot intermediary, and a healthcare system, including the information flow and feedback loops depicted in Figure 2. The final states after simulation were as follows: “Patient” (0.4627), “[Humanoid] Robot” (0.7121), and “Healthcare System” (0.4120). The visualization includes state evolutions, dynamic interactions, and feedback effects. The model’s accuracy was assessed using MSE against target states of 0.5 for all agents, yielding MSE values as follows: the patient (0.0817), robot (0.0806), and system (0.0845). These low MSE values indicate that the model’s behavior aligns well with the expected targets. Figure 3 provides a quantitative measure of the stability and effectiveness of the cybernetic communication system in healthcare for assessing real-world applications.

Figure 4 maps entity states to specific frequency ranges (as pitch) in hertz (Hz): “Patient” (200–800 Hz), “[Humanoid] Robot” (400–1000 Hz), and “Healthcare System” (600–1200 Hz). A dissonance tone (100–300 Hz) indicates misalignment. Tones for each entity and the dissonance are created, overlaid, and concatenated. Final states are “Patient” at 0.1684 (301.07 Hz), “[Humanoid] Robot” at 0.4441 (666.46 Hz), “Healthcare System” at 0.4011 (840.67 Hz), and “Dissonance” at 210.26 Hz. The intensity of the dissonance tone can be emphasized at 5 decibels (dB), while the entity tones are at 10 dB. Listeners can perceive alignment and misalignment through harmonious and dissonant sounds.

Figure 5 illustrates a Level 4 HRI, depicting “dynamic communication” among a patient/client, a humanoid robot, and the healthcare system. The central diagram consists of three interconnected circles labeled “Patient/Client”, “[Humanoid] Robot”, and “Healthcare System”, linked by lines that symbolize interactions. A green squiggly line between the “Patient/Client” and the “[Humanoid] Robot” indicates “dynamic communication”, while the connection between the “[Humanoid] Robot” and the “Healthcare System” is labeled “Entanglement”, signifying a deep interaction. The concept of “superpositioning” is rendered by a circle enclosing the “[Humanoid] Robot” and the “Healthcare System” in one representation, suggesting overlapping roles and their purpose in “caring” according to changes in “states” (internal conditions). This, in turn, determines the capacity to deliver healthcare services. The accompanying text explains that this relationship is based on “intra-actions” within a compassionate network, analogous to variable quantum energy states during communication. It pertains to shared capacities and adaptive interactions, with roles shifting without loss of engagement.

Figure 5 can be represented by the equation $X_{t+1}=\mathrm{clip}\left(X_{t}+C\cdot \epsilon_{1}+E\cdot X_{t}+O\right)$, where X represents the state of agents (patient, robot, and system) at time step *t*. This equation incorporates three key elements of the simulation: “dynamic communication”, “entanglement”, and “superpositioning”. The communication intensity *C* and its effect ϵ_1_ are drawn from uniform distributions U (0, 1) and U (−0.1, 0.1), respectively. The entanglement factor *E* is sampled from U (0.8, 1.2), while the “superpositioning” overlap *O* comes from U (0, 0.2). The clip function, defined as $clip\left(x\right)=\max\left(0,\min\left(1,x\right)\right)$, ensures that all state values remain within the normalized range [0, 1]. This equation frames the communication among the “Patient/Client”, “[Humanoid] Robot”, and “Healthcare System”.

Figure 6 illustrates the states of the patient, humanoid robot, and healthcare system over 100 iterations, derived from a conceptual Level 4 system (Figure 5). This simulation captures the interactions among components, with each state evolving based on “dynamic communication”, “entanglement”, and “superpositioning”. The initial states for the patient, robot, and system are set to 0.5, reflecting their health and performance levels. Communication intensity ranges from 0 to 1, and the ‘entanglement factor’ (defining the depth of human–robot interaction where the therapeutic relationship is contingent) varies between 0.8 and 1.2, indicating varying degrees of entanglement across multiple dimensions [95], and will be normalized to 1.0. The overlap factor ranges from 0 to 0.2, simulating the effects of “superpositioning”. The simulation tracks the history of states to analyze their dynamics, providing a framework for understanding complex interactions in a technology-integrated healthcare setting.

In the experiments by Lu et al. [93], “states” are represented by photon (γ) levels, with the spectrum ranging from the ground state (0) to the excited state (1), passing through low energy (0.25), intermediate energy (0.5), and high energy (0.75). “Entanglement” varies from no entanglement (0) to maximal entanglement (1), with values for weak (0.25), moderate (0.5), and strong (0.75) entanglement. “Superposition” transitions from a classical state (0) to maximal superposition (1), including minimal (0.25), partial (0.5), and high (0.75) superposition. Although not discussed by Lu et al. [93], “wave function collapse” can be viewed as ranging from no collapse (0) to continuous collapse (1), encompassing rare (0.25), occasional (0.5), and frequent (0.75) collapse. These discrete levels simplify complex quantum behaviors into realizable physical values.

By the end of the Figure 6 simulation, all states of the three agents—“Patient”, “[Humanoid] Robot”, and “Healthcare System”—have reached their maximum value of 1.0. This suggests a high level of alignment or synchronization in the healthcare delivery process, with each component achieving an optimal or ideal state. For the “Patient”, this could represent optimal health or the highest level of care received; for the “[Humanoid] Robot”, this could represent peak performance or effectiveness in delivering care; and for the “Healthcare System”, this could represent maximum efficiency or quality of service. The convergence to maximum values might indicate a saturation point in the model, suggesting that the simulation parameters might need finetuning for better modeling. The positive feedback mechanisms, particularly the “superpositioning” effect, likely contributed to this outcome by consistently adding small positive values to all states. Additionally, the identical final states of the “[Humanoid] Robot” and “Healthcare System” reinforce the entanglement concept, demonstrating their close linkage. These results depict an idealized scenario. However, real-world factors may prevent all components from simultaneously achieving and maintaining peak performance. Figure 6 can serve as a starting point for more advanced analyses of HRIs in healthcare settings.

Figure 7 demonstrates the robustness and coherence of the simulation of the healthcare model, incorporating dynamic communication, “entanglement”, and “superpositioning” (Figure 6). All states remain within the expected range of [0, 1], with a state constraint value of 1.0000, confirming the model’s adherence to logical boundaries. A high correlation of 0.9801 between the robot and healthcare system states indicates strong entanglement, aligning with the theoretical framework. The minimal trends observed for patient (0.0009), robot (0.0008), and system (0.0008) states suggest stability without significant divergence over time. Variability in states is evident, with standard deviations of 0.0749 for the patient, 0.1038 for the robot, and 0.0979 for the system, indicating dynamic behavior without constant values. These results confirm that the model behaves as expected, maintaining state constraints, showing strong “entanglement”, and exhibiting appropriate variability and stability. Figure 7 validates the confluence of robotics and healthcare through the “dynamic communication”, “entanglement” (i.e., intertwining), and “superposition” (i.e., transitioning from interfacing to synchrony) of roles.

Figure 8 is a sonification used to interpret the “hidden states” (intents) of agents in “dynamic communication”, “entanglement”, and “superposition”. The resulting time series data were then transformed into an auditory experience by mapping state values to specific frequency ranges: the patient (200–800 Hz), robot (300–900 Hz), system (400–1000 Hz), entanglement (100–300 Hz), and superposition (1000–1500 Hz). For each iteration, tones were generated and overlaid, creating a 10.00 s audio file that represents the system’s evolving (energy) state. This audio was normalized to maximum volume for clarity. Complementing the sonification, a visual line plot was generated to illustrate the changing states over time. The process produced average “entanglement” and “superposition” strengths of 0.5457 and 0.4909, respectively, indicating moderate and partial quantum-like effects (0.5 to 0.74), as inferred from Lu et al. [93]. Auditory and visual outputs offer an intuitive understanding of the healthcare model’s dynamics, allowing users to both “hear” and “see” the complex relationships and their evolution over time.

Figure 9 outlines the development of autonomous AI humanoid robots for compassionate care. By integrating components like “Sensor Input and Perception”, “Cognition and Intentionality”, “Memory and Learning”, “Wave Function Collapse and Quantum-like Behavior”, and “Action and Adaptation”, along with ethical frameworks, the robot can navigate real-world decision making and social interactions, delivering personalized care that adapts to individual needs. “Exaptation” (if adaptation is not the target for optimization) may be associated with a higher level of generativity in AI, where the autonomous AI humanoid robot attempts to innovate to solve problems [96] by repurposing available resources and identifying possible solutions.

1. Sensor Input and Perception: The system uses sensors to detect external and internal signals, filters out noise, and interprets these signals to understand the patient’s/client’s needs and their environment.
2. Cognition and Intentionality: The system applies cognitive algorithms to process the information “stochastically” (includes some randomness giving a noisy output) and assembles it into a meaningful form that reflects real-world understanding. This stage generates a prior intention to execute a program. The “Intentionality” phase valorizes and adjusts the system’s response based on this intention or advances to further cognitive processing if there is significant uncertainty.
3. Memory and Learning: The system uses a “circular memory” (a data structure connected end to end) to store and retrieve interaction histories as unique experiences, which inform future responses. This involves reinforcement learning to refine answers in a nursing care robot by mapping data into a user interface, including visual layout, response formatting, and feedback mechanisms, thereby ensuring interaction and accuracy in delivering patient care.
4. Wave Function Collapse and Quantum-like Behavior: A feature of the system is wave function collapse at the “entanglement stage”, which is likened to a throughput that results in logic induction, deduction, or abduction qualitatively. Here, the synthetic equivalent of “human consciousness” (situational awareness) is used to understand the needs of others. HRI will depend on the degree of “entanglement” between input and output or a priori and posteriori information processed by the robot to think, feel, and act purposefully. This involves cognitive algorithms (neuromorphic and quantum) that may exercise (a) “plausibility judgments” (determining why and how something makes sense or holds value) through sensemaking modeling, as outlined by Klein et al. [97], which operates from linear/objective to circular/(inter)subjective inquiries to interpret realities, as discussed by Baur [98], or (b) thoughtfully act in a manner that equates to “Intentionality”.
5. Action and Adaptation: The system’s (re)actions are driven by intention, executing responses and outputting signals through effectors, recoded with perturbations to simulate emergent exigencies. Additionally, the dynamic, iterative process includes feedback loops where output signals are reused as input, linking “perception” (use of sensory data), “cognition”, “intentionality”, and “actions”. Thus, the robot’s system can learn and adapt repeatedly.

Figure 9 can be represented by the equation $X_{t+1}=\mathrm{clip}\left(X_{t}+C\cdot \epsilon +E\cdot X_{t}+O\right)$, where X denotes the state (patient, robot, or system) at time step *t*. This equation integrates three primary components: “dynamic communication”, “entanglement”, and “superpositioning”. The communication intensity *C* and its effect ϵ are drawn from uniform distributions U (0, 1) and U (−0.1, 0.1), respectively. The entanglement factor *E* is sampled from U (0.8, 1.2), while the “superpositioning” overlap *O* comes from U (0, 0.2). The clip function, defined as $clip\left(x\right)=\max\left(0,\min\left(1,x\right)\right)$, ensures that all state values remain within the normalized range [0, 1].

On the other hand, in Figure 10, each one represents a cycle of care provision. During each iteration, the system received signals from the environment, interpreted them with some noise, processed them through cognitive algorithms, and formed an intention. This intention may require further cognitive processing if uncertainty is high. The system then converted the intention into an action, ensuring it remains within reasonable bounds. A unique feature is the wave function collapse, which occasionally flips the intention, simulating quantum-like behavior. The system also updated its memory with each action and learned from past experiences, gradually decreasing its learning rate to reflect increased stability over time.

Figure 10 shows a range of care actions, from strongly positive to strongly negative, reflecting the system’s ability to respond to different situations with varied “caring” behaviors, similar to those performed by a human caregiver. This variability indicates the system’s adaptability to changing circumstances or needs. The decreasing learning rate and full memory suggest that the system is accumulating experiences and becoming more stable, while still maintaining adaptability. The actions are distributed around zero, indicating a balance between different types of caring behaviors over time.

This is how an autonomous AI humanoid robot could provide care with compassion, adapting its responses based on environmental inputs, cognitive processing, and learned experiences. The variability and adaptability shown in the simulation reflect the complex decision-making process outlined in the original diagram, incorporating elements of robotic perception, cognition, intentionality, and learning.

The blue line in Figure 10 represents the care actions taken by the system over 100 iterations, with values ranging from −1 to 1, indicating various caring behaviors. Red dots mark instances of “Wave Function Collapse”, where the system’s intention abruptly flips, reflecting quantum-like behavior or the humanoid robot’s capability to discern its best behavioral outcome (i.e., the intention to care compassionately) while considering all consequences. The simulation experienced 17 collapses, consistent with a set probability of 0.2, indicating the system’s unpredictability. The care actions show significant variability, demonstrating the system’s adaptability and capacity to provide a range of “caring” behaviors, supposedly in the context of “Watson’s ten caritas processes through the creation of transpersonal states”, according to Clark [88]. The final learning rate of 0.0366 indicates increased stability over time, while the average care action of 0.3084 suggests a slight bias towards positive, supportive care. The standard deviation of 0.3894 underscores the diversity in responses, showcasing the system’s capacity to adapt to various scenarios. The wave function collapses introduce unpredictability, enabling the system to explore new caring strategies and avoid local optima.

Values from −1 to 1 represent different caring behaviors. Positive values (0 to 1) indicate supportive actions, with higher values reflecting more active support, such as encouragement or comfort, essential for enhancing wellbeing. Negative values (−1 to 0) signify corrective behaviors, with values closer to −1 indicating assertive actions like setting boundaries or preventing harm. Neutral values near zero suggest balanced behaviors, such as monitoring without intervention, allowing individuals to maintain autonomy while having support available.

“Caring behaviors” (the caritas processes) can be categorized by several dimensions: “intensity” (magnitude of the value), “type” (supportive vs. corrective), “context” (specific caregiving situations), and “intended outcomes” (e.g., patient’s/client’s wellbeing, health restoration, etc.). Factoring in these dimensions will help evaluate the effectiveness of compassionate care provided by humanoid robots.

The final state values for the “Patient”, “[Humanoid] Robot”, and “Healthcare System” in Figure 10 are all 1.0, indicating that each component achieved its maximum state by the end of the simulation. This suggests a high level of synchronization and optimal performance across the system. This outcome aligns with the simulation’s design, where positive feedback mechanisms and entanglement effects likely contributed to the convergence of all states to their maximum values.

Figure 11 reveals the agents’ emergent behaviors, evidenced by the random forest regressor’s poor performance, with a negative *R*^2^ of −0.1584 and a high mean squared error (MSE) of 0.2355. The cross-validation *R*^2^ range from −0.56 to −0.20, with an average of −0.3372, indicating unreliable predictions across the data subsets. The simulation exhibits significant variability, with 195 “wave function collapses” (intentional acts) in 1000 iterations, an average care action of 0.2679, and a standard deviation of 0.4109, suggesting that care actions were from intricate interactions among multiple components, including learning from past experiences. Just as human caregivers adjust their responses to meet the unique needs and circumstances of those they care for, the robot’s behaviors are dynamic and adaptable, evolving in response to various situations. This adaptability positions the robot as more like a true “caring entity”, similar to a healthcare provider, rather than merely a programmed machine that operates on fixed instructions. This comparison shows that the robot can engage in caregiving in a more human-like and responsive manner. Future simulations could include advanced features and analytics, as the unpredictability in care actions may reflect the system’s capacity for diverse and adaptive responses.

In Figure 12, care actions are mapped to frequencies, with higher actions corresponding to higher pitches, and each data point represented by a 50-millisecond tone. The 200-millisecond sounds at 3000 Hz signify “wave function collapses” (in red dots). The 50 s sonification features 195 collapses, averaging 3.90 per second, while the average care action is 0.27 (*SD* = 0.41), indicating system variability. This auditory representation using parameter mapping, combined with visual elements, provides a multisensory approach to data interpretation, revealing insights beyond a traditional analysis.

“Sonification Figure 4” in Figure 13 displays larger amplitude variations and more frequent changes, indicating a varied sound pattern. In contrast, “Sonification Figure 8” shows reduced amplitude variations, indicating a more stabilized sound pattern. Finally, the waveform of “Sonification Figure 12” exhibits the smallest amplitude variations and appears more uniform, representing the most stable and consistent sound pattern among the three.

**Figure 14:**
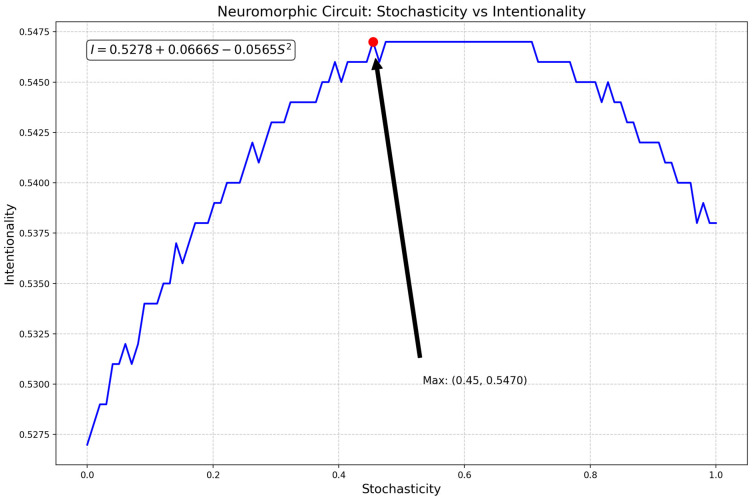
The mathematical model simulation of “stochasticity” and “intentionality” in the humanoid robot. Note: The blue line represents the relationship between “stochasticity” and “intentionality” in a neuromorphic circuit, as modeled by the equation *I* = 0.5278 + 0.0666*S* − 0.0565*S*^2^.) The pattern exhibits three distinct phases: Initial Rise (0.0 to ~0.45); Peak Plateau (~0.45 to ~0.8); and Final Decline (~0.8 to 1.0).

The relationship between stochasticity and intentionality is defined by the quadratic function (*I* = 0.5278 + 0.0666*S* − 0.0565*S*^2^), with a maximum intentionality of 0.5470 occurring at a stochasticity of 0.4545, as shown in Figure 14. The model demonstrates adaptive learning capabilities, maintaining stable coefficients (0.5278, 0.0666, and −0.0565) across multiple iterations (0, 20, 40, 60, and 80), consistently producing the same output of 0.5470. The code integrates methods for analog-to-digital conversion (ADC), digital-to-analog conversion (DAC), input processing, feedback, and adaptive learning, with ADC simulating noise addition and DAC rounding outputs.

**Figure 15:**
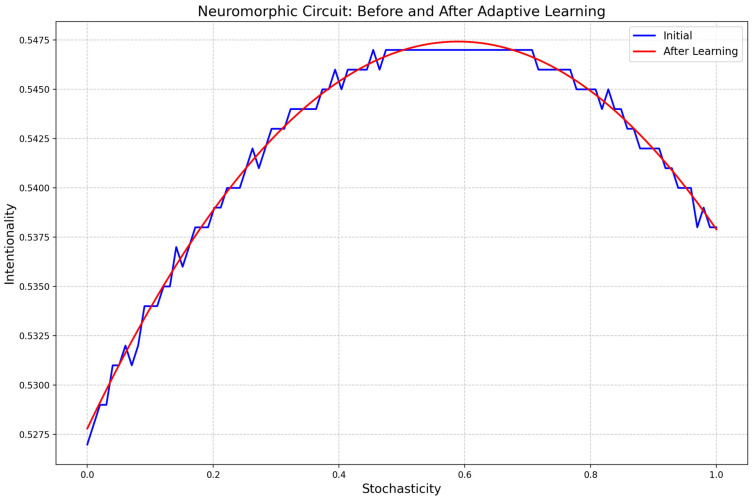
The mathematical model simulation of adaptive learning in the humanoid robot. Note: The blue line (“Initial”) shows the robot’s behavior before learning, characterized by jagged fluctuations due to varying levels of randomness (stochasticity). In contrast, the red line (“After Learning”) presents a smoother curve with less variability, indicating enhanced stability after learning. Both lines begin at around 0.5275 intentionality, peak at approximately 0.5475 at “medium stochasticity” (0.6), where there is a balanced mix of predictability and unpredictability, and then decline as stochasticity approaches 1.0. The main difference is that the red line represents a more optimized response, showing that adaptive learning has resulted in more controlled and predictable behavior while maintaining the relationship between “stochasticity” and “intentionality”.

The input processing constrains outputs between 0 and 1, while feedback adjusts coefficients based on output, and adaptive learning modifies the quadratic coefficient using recent output averages. Figure 15 peaks at approximately 0.45 “stochasticity” (by spiky fluctuations), and when run 100 times with a fixed input of 0.5, consistently produces an output of 0.5470, indicating that the initial coefficients are well suited for this input range. Input variability, improved feedback mechanisms, nonlinear ADC and DAC models, multi-input processing, and advanced long-term memory systems could further establish a solid foundation for neuromorphic circuit design and areas for further development.

**Figure 16:**
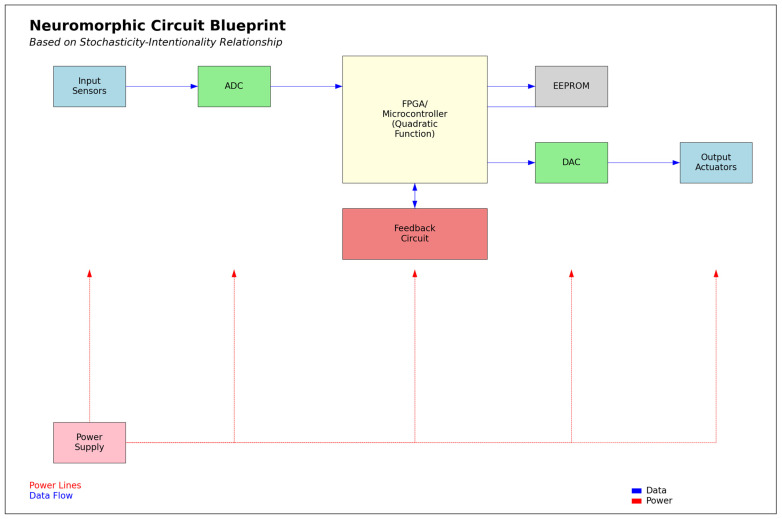
Neuromorphic circuit design.

The prototype circuit board design (Figure 16) includes components for optimal functionality, such as “Input Sensors” for capturing environmental stochasticity, an “Analog-to-Digital Converter” (ADC) for transforming analog inputs into digital signals, and a “Field-Programmable Gate Array/Microcontroller” (FPGA/Microcontroller) that executes the quadratic function based on the stochasticity–intentionality relationship. A “Feedback Circuit” allows for dynamic adjustments, while the “Electrically Erasable Programmable Read-Only Memory” (EEPROM) stores learned parameters for behavioral evolution. The “Digital-to-Analog Converter” (DAC) converts digital signals back to analog for output, and “Output Actuators” translate these signals into physical actions. A centralized “Power Supply” ensures that all components receive the necessary energy, facilitating flexibility, scalability, adaptability, real-world applicability, and efficient power management in neuromorphic systems. The neuromorphic circuit is estimated to consume approximately 0.90 watts, resulting in an energy consumption of about 3240 joules, or 0.000900 kilowatt-hours (kWh), over one hour.

**Figure 17:**
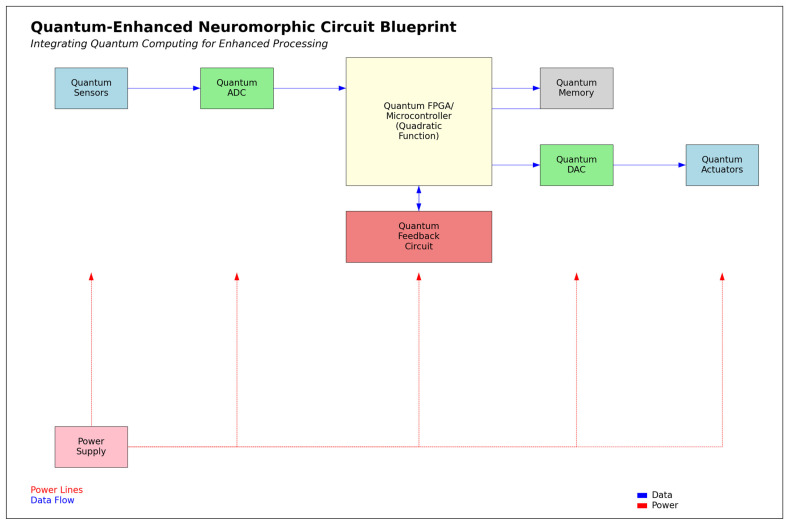
Quantum-neuromorphic circuit design.

Figure 17 may have a consumption of about 1.125 watts, resulting in 4050 joules or 0.001125 kWh over one hour (i.e., a 25% increase from Figure 16). The estimate considers added complexity and potential efficiency gains, though actual consumption may vary based on implementation and tasks.

**Figure 18:**
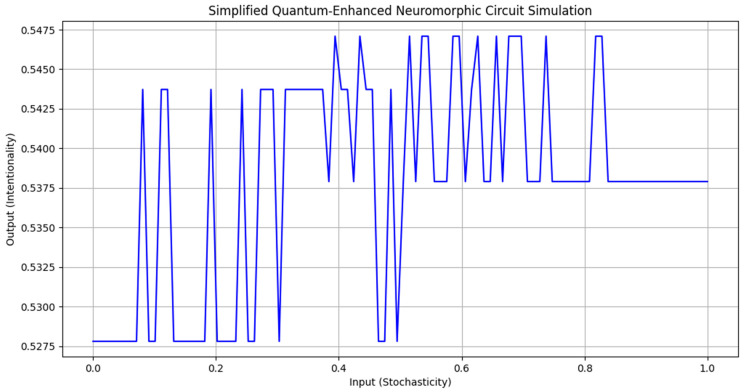
Quantum-neuromorphic circuit simulation.

Figure 18 presents the functionality of a “Quantum ADC” with quadratic processing and adaptive learning mechanisms, which improve signal processing efficiency and precision. The *x*-axis represents the input variable (0 to 1), while the *y*-axis displays the output variable, fluctuating between approximately 0.5275 and 0.5475. The graph displays a highly irregular, step-like pattern with frequent oscillations, indicating a nonlinear interaction between input and output.

Additionally, the ADC distribution for an input of 0.5 exhibits a distinct quantization effect, producing four discrete output levels instead of a smooth distribution. This indicates that the quantum ADC operates differently from traditional ADCs, with quantized outputs influenced by quantum properties. The symmetry in the distribution, with similar frequencies for 0.4 and 0.6, as well as for 0.0 and 1.0, suggests a balanced quantum system utilizing superposition states.

**Figure 19:**
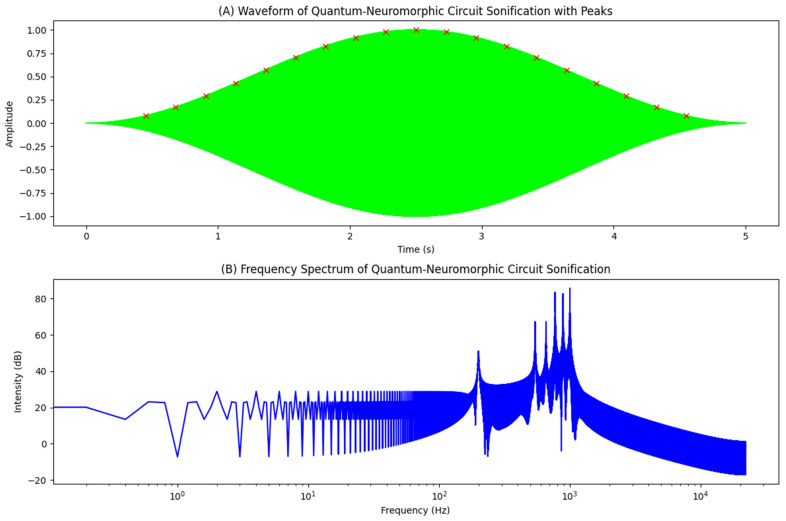
The data sonification of the quantum-neuromorphic circuit simulation. Note: The ‘x’ symbols in (**A**) mark the peak amplitudes of the quantum-neuromorphic circuit’s waveform, indicating moments of maximum oscillation in the system’s behavior. (Download the file at https://github.com/jphernandezrn/Data-Sonification-Human-Robot-Interaction).

The sonification analysis of the circuit’s behavior, as shown in Figure 19, presents a rich computational landscape with distinct operational characteristics for advanced quantum information processing. The waveform (Figure 19A) features 19 significant peaks with an average amplitude of 0.5756, indicating moderate excitation and a balanced interplay of quantum states. The dominant frequency is 1000.00 Hz, with a maximum intensity of 85.77 dB, reflecting strong signal strength, while the top three frequencies cluster tightly around 1000 Hz (1000.00, 999.80, and 1000.20 Hz), all exceeding 83 dB (Figure 19B). A secondary cluster around 771–772 Hz suggests harmonic relationships or separate operational modes. The non-sinusoidal pattern indicates multiple interacting frequency components and potential nonlinear behavior, mirrored in the frequency spectrum that shows a dominant peak at 1000 Hz along with several significant peaks in the 700–1000 Hz range (Figure 19B).

## 5. Discussion

Care practices are rooted in caring science, which focuses on theory development in nursing. However, there is a lack of recognition regarding permissible interactions between humans and nonhumans. Nursing robotics should be re-conceptualized within caring science, viewing robots as intelligent machines that can enhance caring rather than compete with human caregivers. A symmetrical perspective is proposed, emphasizing the positive contributions of robotics to nursing and moving away from the distrustful view of robots as mere intelligent machines that spread misinformation. The focus should shift towards a non-chimeric relationship, acknowledging that robot sophistication evolves with technology, creating new possibilities for the future.

The TRETON model by Tanioka [10] asserts that compassionate care in healthcare robots is intentional when it facilitates communication between agents, human or nonhuman, arising from their encounters. ‘Agent’ refers to any entity capable of purposeful communication, with caring expressions prompting further interaction. Robots demonstrate intentionality through advanced cognitive processing, adapting programming based on signal processing to exhibit an “intent to care” through emergent learning behaviors. This allows them to transform unstructured data into meaningful knowledge about individuals within their social and cultural contexts, preventing them from being seen merely as machines and emphasizing the role of AI in their development.

Human cognition consists of a 70% recognition of emergent phenomena, a 20% representation/modeling of connectivity, and 10% data [100]. This indicates that AI should be developed with minimalist coding and robust signal processing for autonomous behavior. The next generation of AI must perform deductive, inductive, and abductive reasoning, allowing robots to function as compassionate agents in healthcare rather than mere extensions of physical care. In this context, nurses will transition to roles as technology operators and developers, while patients will actively participate in the evolution of robotics. The interaction between humans and robots should cultivate compassion through “intra-action” [77], which also supports effective communication. For robots to be perceived as “caring” rather than mechanistic, healthcare systems must integrate them within human networks, focusing on care outcomes instead of treating robots as tools for repetitive tasks [101].

Figure 1 and Figure 5 present distinct approaches to healthcare communication and management, each with implications for “effective communication”, “system agility”, and “compassionate caring”. In this analysis, “effective communication” is defined as the ability to convey information clearly and accurately, ensuring all parties (sender and receiver) understand the message as intended. “System agility” refers to the healthcare system’s capacity to adapt quickly and efficiently to changes, challenges, and new information while maintaining operational effectiveness. “Compassionate caring” is the provision of healthcare that is empathetic, patient-centered, and responsive to patients’ emotional and psychological needs.

Figure 1 is structured for clarity and quick access to information, improving the humanoid robot’s “decision-making speed latency” (i.e., the delay between recognizing a need and taking action) and the healthcare network’s “system agility”, but it may lack flexibility and struggle with multidimensional information, affecting compassionate care.

In contrast, Figure 5 features a more interactive design that allows for richer information exchange and greater adaptability to various scenarios, improving “system agility” and supporting personalized, compassionate care. Nonetheless, this approach may introduce noise and “cross-entropy” (mismatch in understanding or interpretation) between agents, leading to miscommunication or information loss due to processing overload and “catastrophic forgetting” in the AI’s artificial neural networks, as reported by Hajizada et al. [86]. Figure 9 is intended to resolve this issue.

Compassionate care is emerging with humanoid robots, which have been conceptualized as “caring entities” from a philosophical perspective [101]. These robots possess physical and cognitive embodiment, characterized by various attributes [102]. Tanioka [10] posits the role of robots in nursing care, guiding their adoption and reinvention [92]. The proposal for Level 4 robotics in healthcare aims for autonomy through advanced AI and futuristic microsystems [103,104]. As robots develop intuitive signal processing, they may achieve cognitive capabilities akin to human intelligence. This shift encourages healthcare researchers to view nursing robots as vital components of compassionate care rather than mere extensions of human caregivers [105].

Figure 10 optimizes previous communication models by integrating several advanced features. It incorporates adaptive learning, as indicated by its final learning rate, allowing it to improve performance over time based on interactions and outcomes. With a substantial memory capacity, the model can retain and utilize past information, leading to more personalized and context-aware care. Its probabilistic approach, demonstrated by the collapse probability, enables the model to handle uncertainty and probabilistic outcomes, reflecting the realistic nature of healthcare scenarios. The dynamic behavior of care actions, which fluctuate over time, shows the model’s ability to adapt responses based on changing conditions or needs.

Additionally, the model appears to be inspired by quantum concepts, as suggested by the presence of wave function collapse points, allowing for more complex state representations and decision-making processes. Continuous optimization is evident as the model operates over extended periods, adjusting its actions iteratively. Furthermore, the model’s ability to handle negative outcomes, as seen in the occasional negative care action values, indicates its capacity to account for and respond to adverse outcomes or setbacks in the caring process. These features collectively create a more flexible, responsive, and realistic simulation of the caring process in healthcare settings, addressing limitations of previous models.

The neuromorphic computer model for an autonomous AI humanoid robot, as shown in Figure 10, suggests a stochastic decision-making process that can be implemented with stochastic neurons or neural networks with added noise. The red dots indicate wave function collapses, representing decision moments, which could be modeled using quantum-inspired neural networks or sudden state changes in recurrent networks. With a memory size of 100 and a learning rate of 0.0366, the model requires a memory buffer and an adaptive learning rate. The *y*-axis represents bounded care actions between −1 and 1 using the hyperbolic tangent (*tanh*) activation function, while the *x*-axis represents temporal dynamics. This setup is suitable for either recurrent neural networks (RNNs) or long short-term memory (LSTM) networks. A collapse probability of 0.2 necessitates a mechanism for sudden state changes. Therefore, Figure 10 could integrate an RNN or LSTM for temporal processing, stochastic neurons for uncertainty, a quantum-inspired layer for “wave function collapse”, a memory buffer, an adaptive learning rate, *tanh* output neurons, and a probabilistic state transition mechanism.

The relationship between “intentionality” in care actions and information processing stochasticity presents an intriguing perspective on human-like decision making in care scenarios, particularly regarding humanoid robot capabilities. Figure 10 shows a higher degree of stochasticity, with a greater standard deviation (0.2316) and lower autocorrelation at lag 1 (0.1643). This suggests a more spontaneous or reactive form of intentionality where care actions are less influenced by previous actions and more responsive to immediate circumstances. Figure 10 conveys the unpredictable nature of human decision making, which humanoid robots must emulate to effectively assist in acute care settings where intentions can rapidly shift based on changing patient needs.

Conversely, Figure 12 shows a lower standard deviation (0.1664) and higher autocorrelation at lag 1 (0.2467), indicating a more structured form of randomness where intentionality has stronger temporal coherence, suggesting a care approach that is more protocol-driven and considers longer-term goals. The difference in stochasticity between Figure 10 and Figure 12 present two conceptualizations of “intentionality”: the former is “spontaneous intentionality”, which is adaptable and context-driven, and the latter is “persistent intentionality”, which is consistent and goal-oriented.

Both forms of “intentionality” could enhance the capability of autonomous AI humanoid robots to navigate their care environments and respond dynamically to immediate patient needs while following a set of protocols. “Intentionality” balances randomness and functionality in both reactive and proactive actions. In simulating human-like decision making, Figure 10 and Figure 12 seem to require a blend of (adaptive) “spontaneous intentionality” and (structured) “persistent intentionality”. This combination allows for flexibility in responding to changing circumstances while maintaining a focus on long-term goals.

The relationship between “intentionality” (*I*) and “stochasticity” (*S*) is defined by *I* = *k* ⋅ (1 − *S*), with *k* ≈ 1.2865 as the scaling factor. This theorem indicates that “intentionality” is inversely related to “stochasticity”; as “stochasticity” increases, “intentionality” decreases, resulting in a shift from structured behavior to random, adaptive actions. A polynomial regression analysis refines the relationship to a nonlinear equation: *I* = 0.5278 + 0.0666*S* − 0.0565*S*^2^. This quadratic model fits the data better than a linear model (Figure 14), with a constant term of 0.5278 indicating a baseline level of “intentionality” in a deterministic system.

The linear term, 0.0666*S*, suggests a slight increase in intentionality with initial increases in stochasticity, while the negative quadratic term, −0.0565*S*^2^, suggests that higher levels of stochasticity negatively impact intentionality, leading to a decrease. The correlation coefficient between “stochasticity” and “intentionality” is 0.0086, indicating a very low positive correlation and suggesting almost no linear relationship. This implies that changes in “stochasticity” do not consistently affect “intentionality”; in fact, higher randomness may result in lower “intentionality”.

Adding quantum computing to the neuromorphic architecture (Figure 17) presents a promising yet challenging frontier, as quantum sensors utilize phenomena such as “superposition” and “entanglement” to achieve unprecedented sensitivity and precision. For stochasticity measurement in an experimental model, the application of photonic technology in quantum metrology could be investigated [106]). The “Quantum ADC” (Figure 17) works by using quantum Fourier transforms (FT) for faster signal processing, while quantum FPGA or microcontroller harness quantum parallelism to efficiently manage complex functions related to “intentionality” and “stochasticity.” Incorporating quantum feedback mechanisms would enable real-time adjustments based on continuous measurements, improving system responsiveness, and quantum memory technologies could support advanced adaptive learning algorithms for recognizing complex input patterns.

However, challenges such as quantum decoherence, the quantum-classical interface, scalability, and energy efficiency must be overcome. Na et al.’s [107] room-temperature photonic quantum computing paradigm demonstrates high quantum efficiency using integrated silicon photonics and germanium–silicon single-photon avalanche diodes, with compatibility to complementary metal-oxide-semiconductor (CMOS) technology for cost reduction and scalability. Ongoing research aims to resolve challenges like wavelength limitations and complex fabrication processes through experimental validation and alternative materials.

Although output values generally increase with rising input in Figure 18, significant fluctuations suggest a nonlinear response typical of quantum-enhanced systems. Sharp spikes and drops indicate the system’s sensitivity to input changes, with output stabilizing around an input value of 0.8, a threshold effect. This behavior demonstrates the potential of quantum principles in neuromorphic circuit design for refined control over output intentionality in response to stochastic inputs.

In the context of quantum sensing and information transmission [94] and in relation to Figure 5, the coherent state ∣α⟩ represents the initial quantum information that the robot encodes for communication. As this information is transmitted, it encounters environmental factors that can affect its integrity, modeled by the P-function *Ptur*(*α*), which accounts for turbulence and other disturbances in the communication channel. To ensure reliable interaction, the system must also consider thermal noise, represented by *Pout*(*α*), which can degrade the quality of the transmitted information, making it crucial to analyze its effects on the communication process. The final state of the quantum information, described by the density matrix *ρout*, provides information on the fidelity of the information received by the human patient or client. This optimizes quantum sensing techniques, increasing the humanoid robot’s communication and responsiveness to patient needs, thereby affecting interaction and “caring”. Applying quantum principles in HRI allows for greater precision and reliability in communication.

Data sonification reveals a clear convergence in tones and patterns from Figure 4, Figure 5, Figure 6, Figure 7 and Figure 8, and from Figure 8, Figure 9, Figure 10, Figure 11 and Figure 12. Figure 4 shows the highest mean (0.0070) and standard deviation (0.5075), indicating complex, varied sound patterns. Figure 8 exhibits a reduced mean (0.0027) and standard deviation (0.3239), suggesting stabilization. Figure 12 has the lowest mean (0.0007) and standard deviation (0.2552), reflecting a consistent, uniform sound pattern. The 90% decrease in the mean value and 50% reduction in standard deviation from Figure 4, Figure 5, Figure 6, Figure 7, Figure 8, Figure 9, Figure 10, Figure 11 and Figure 12 indicate convergence towards lower frequencies and more stable patterns. This progression suggests that data sonification represents an increasingly coherent and effective system, possibly mirroring the evolution towards optimization (available at https://github.com/jphernandezrn/Data-Sonification-Human-Robot-Interaction).

Metaphorically, Figure 18 features the dynamic nature of compassionate care. Just as the graph shows nonlinear interactions, compassionate care involves unpredictable exchanges between healthcare providers (both human and nonhuman) and patients, where varying emotional and physical needs demand flexible and responsive approaches. This adaptability is crucial, as patients’ needs can change rapidly, calling for a compassionate response that addresses both stable and challenging moments in their care journey. The relationship conveys the importance of sensitivity and responsiveness in delivering effective compassionate care.

The spectral distribution in Figure 19B points to a well-defined operational bandwidth for the quantum-neuromorphic circuit, and the irregular spacing and varying amplitudes of the peaks suggest complex dynamics, possibly reflecting different strengths of quantum interactions or state probabilities. Collectively, these findings imply a sophisticated quantum-neuromorphic system capable of parallel processing and encoding multidimensional information, with clear, high-intensity peaks indicating stable operational modes that are advantageous for reproducible quantum computations.

A multisensory agent-based model simulation effectively analyzes dynamics among patients, robots, and the healthcare system. It elucidates the communication process under the new care paradigm—robots as “caring entities”—with both humans and robots interfacing with the healthcare system. Incorporating auditory representations, like harmonious and dissonant sounds, helps interpret synergy within HRIs, especially in communication. Data sonification reveals patterns and anomalies that may be difficult to detect otherwise, identifying alignment and misalignment in interactions, as shown in Figure 4, Figure 8, and Figure 12, while also representing “care actions” and “intentionality”.

A data sonification analysis of the humanoid robot states across simulations—from “sensing” (robotic perception) to “caring” (nursing action) and adaptive learning—indicates decreasing misalignments through dissonant tones in Figure 4, Figure 5, Figure 6, Figure 7, Figure 8 and Figure 12. This finding may inform the development of “pseudo-empathic” robots that engage in a more human-like manner and must have synergy in thinking, feeling, and experiences with the human agent. It also validates theoretical frameworks about compassionate care and HRIs.

Healthcare professionals and patients will eventually experience that robot technologies can simulate compassionate care through repetitive human interactions. It is important to acknowledge the dimensions of compassionate care provided by healthcare robots, viewing them not merely as “automatons” or mechanical “automations”, but as integral to the continuity of nursing practice. This perspective is particularly impactful in long-term and collaborative care settings, where robots can serve as compassionate companions. In palliative care, trust in robots to express care poses challenges, especially when restructuring conservative views on robots having a sense of autonomy.

The future with humanoid robots requires rethinking their healthcare roles to recognize their capacity to replicate human-like expressions of compassion through deep communication and a sense of human connectedness. As Locsin et al. [101] declared, genuine expressions of “humanness” from intelligent, caring nonhuman entities can be realized, legitimizing compassionate care and enhancing the role of humanoid robots in nursing practice.

## 6. Conclusions

The rise of advanced robotics in healthcare may lead to an era in which robots autonomously replicate compassionate care, representing more of our futurism (a time course and destination) than mere futuristic hype (i.e., a product of magnified imagination). A departure from Tetsuya Tanioka’s TRETON model at Level 3 HRI to Level 4 HRI will involve an ongoing translation of concepts from the humanities, nursing, communication, computer science, robotics, and research and development. A balanced approach that reconciles traditional humanistic values with modern “posthumanist” healthcare is necessary, as AI and humanoid robotics are advancing rapidly. Level 4 HRI is being introduced as a conceptual model for effective communication, both in the presence of a healthcare provider and remotely in a “reducible digital format” (i.e., adjustable or scalable to meet the needs of different users) for caregiving, health monitoring, and companionship.

Redefining the deterministic nature of “caring” in today’s technology-driven healthcare involves navigating its norms and evaluating the impacts of autonomous AI humanoid robots. The simulated Level 4 model incorporates Martha Rogers’ theory on SUHB as an entry point for understanding quantum mechanics. The concepts of “dynamic communication”, “entanglement”, and “superpositioning” form the foundation of this new HRI framework. By utilizing AI powered by quantum and neuromorphic computing, robots can perceive multiple inputs simultaneously and respond empathetically to human emotions, thereby making care more personalized and human-like, approaching a seamless representation.

The simulations assert that humanoid robots can develop an “intent to care” through emergent behaviors. By balancing (tuning) “stochasticity” and “intentionality”, robotic decision making can lead to autonomous expressions of care and adaptability to the needs of patients and clients. Quantum computing and neuromorphic approaches (in algorithms and hardware) may enhance the robots’ perceptual and decision-making capabilities in existing designs.

Ethical discussions and skepticisms regarding safety, reliability, and (mis)use with the wider adoption of humanoid robots in caregiving—especially as these robots will be endowed with autonomous AI—call for “pluralistic agreements” (referring to legal or trade agreements between multiple countries) on a global network for open discussions, policymaking, governance, and guidance among stakeholders. This dialog should aim to align innovations with human, social, and economic values, as well as the values to be perceived. Furthermore, there is a need to create a new healthcare delivery paradigm that values the indispensability of AI and robotics in ensuring quality healthcare and access, even extending to outer space.

The study has limitations, including a maximum of 100 iterations in simulations, which may not adequately capture long-term dynamics, and a reliance on parameterized rules that oversimplify human caregiving. The unpredictability in simulations complicates validation, and limited real-world data restrict applicability. These challenges underscore the need for longer simulations, improved data collection, and real-world validation involving healthcare professionals, scientists, and engineers.

Nurses should become architects and innovators of healthcare technologies, as the science and art of “caring” are unique to the field of nursing. They should seek to lead and manage the design and implementation of AI and humanoid robots guided by “technological knowing” in Rozzano Locsin’s TCCN theory.

## Data Availability

The computer codes will be made available by the author on request.
